# Supplementary material for: A method yielding comparable estimates of the fraternal birth order and female fecundity effects in male homosexuality
Source: Proc Biol Sci. 2020 Mar 18;287(1923):20192907. doi: 10.1098/rspb.2019.2907 (PMC7126035; doi:10.1098/rspb.2019.2907)
Supplement: A Method Yielding Comparable Estimates of the Fraternal Birth Order and Female Fecundity Effects in Male Homosexuality [file rspb20192907supp1.pdf]

# Supplementary Material

Ray Blanchard, Jurian Krupp, Doug P. VanderLaan, Paul L. Vasey, and Kenneth J. Zucker.

A Method Yielding Comparable Estimates of the Fraternal Birth Order and Female Fecundity Effects in Male Homosexuality.

Proceedings of the Royal Society B. doi: 10.1098/rspb.2019.2907

1. Means and standard deviations of computed variables

2. Correlations between computed variables

## Descriptive Statistics

| | Mean | Std. Deviation | N |
|---|---|---|---|
| Percent Loss | 31.9815 | 18.37927 | 14 |
| Proportion of Homosexual Subjects | .3630 | .20478 | 14 |
| Proportion of Only Children | .4867 | .10227 | 14 |
| FBOE | 1.5190 | .91556 | 14 |
| FFE | 1.0820 | .47056 | 14 |
| $Odds_{11}$ | .7519 | .62767 | 14 |
| $Odds_{12}$ | .6689 | .56507 | 14 |
| $Odds_{22}$ | .9190 | .69640 | 14 |

## Correlations

| | | Percent Loss | Proportion of Homosexual Subjects | Proportion of Only Children | FBOE | FFE | $Odds_{11}$ | $Odds_{12}$ | $Odds_{22}$ |
|---|---|---|---|---|---|---|---|---|---|
| Percent Loss | Pearson Correlation | 1 | -.154 | -.849** | .105 | -.518 | .038 | -.348 | -.285 |
| | Sig. (2-tailed) | | .599 | .000 | .721 | .058 | .898 | .223 | .323 |
| | N | 14 | 14 | 14 | 14 | 14 | 14 | 14 | 14 |
| Proportion of Homosexual Subjects | Pearson Correlation | -.154 | 1 | .193 | .052 | -.459 | .933** | .888** | .923** |
| | Sig. (2-tailed) | .599 | | .509 | .859 | .099 | .000 | .000 | .000 |
| | N | 14 | 14 | 14 | 14 | 14 | 14 | 14 | 14 |
| Proportion of Only Children | Pearson Correlation | -.849** | .193 | 1 | -.153 | .430 | .023 | .347 | .244 |
| | Sig. (2-tailed) | .000 | .509 | | .600 | .125 | .938 | .224 | .400 |
| | N | 14 | 14 | 14 | 14 | 14 | 14 | 14 | 14 |
| FBOE | Pearson Correlation | .105 | .052 | -.153 | 1 | -.445 | -.039 | -.202 | .236 |
| | Sig. (2-tailed) | .721 | .859 | .600 | | .110 | .894 | .489 | .416 |
| | N | 14 | 14 | 14 | 14 | 14 | 14 | 14 | 14 |
| FFE | Pearson Correlation | -.518 | -.459 | .430 | -.445 | 1 | -.528 | -.099 | -.344 |
| | Sig. (2-tailed) | .058 | .099 | .125 | .110 | | .053 | .737 | .229 |
| | N | 14 | 14 | 14 | 14 | 14 | 14 | 14 | 14 |
| $Odds_{11}$ | Pearson Correlation | .038 | .933** | .023 | -.039 | -.528 | 1 | .799** | .819** |
| | Sig. (2-tailed) | .898 | .000 | .938 | .894 | .053 | | .001 | .000 |
| | N | 14 | 14 | 14 | 14 | 14 | 14 | 14 | 14 |
| $Odds_{12}$ | Pearson Correlation | -.348 | .888** | .347 | -.202 | -.099 | .799** | 1 | .880** |
| | Sig. (2-tailed) | .223 | .000 | .224 | .489 | .737 | .001 | | .000 |
| | N | 14 | 14 | 14 | 14 | 14 | 14 | 14 | 14 |
| $Odds_{22}$ | Pearson Correlation | -.285 | .923** | .244 | .236 | -.344 | .819** | .880** | 1 |
| | Sig. (2-tailed) | .323 | .000 | .400 | .416 | .229 | .000 | .000 | |
| | N | 14 | 14 | 14 | 14 | 14 | 14 | 14 | 14 |

**. Correlation is significant at the 0.01 level (2-tailed).
